# Epidemiology, Phylogeny, and Evolution of Emerging Enteric *Picobirnaviruses* of Animal Origin and Their Relationship to Human Strains

**DOI:** 10.1155/2014/780752

**Published:** 2014-07-17

**Authors:** Yashpal S. Malik, Naveen Kumar, Kuldeep Sharma, Kuldeep Dhama, Muhammad Zubair Shabbir, Balasubramanian Ganesh, Nobumichi Kobayashi, Krisztian Banyai

**Affiliations:** ^1^Division of Biological Standardization, Indian Veterinary Research Institute, Izatnagar, Uttar Pradesh 243122, India; ^2^Division of Pathology, Indian Veterinary Research Institute, Izatnagar, Uttar Pradesh 243122, India; ^3^Quality Operations Laboratory, University of Veterinary and Animal Sciences, Lahore 54600, Pakistan; ^4^National Institute of Cholera and Enteric Diseases, P-33, C.I.T. Road, Scheme-XM, Beliaghata, Kolkata, West Bengal 700 010, India; ^5^Department of Hygiene, Sapporo Medical University School of Medicine, S-1 W-17, Chuo-Ku, Sapporo 060-8556, Japan; ^6^Veterinary Medical Research Institute, Hungarian Academy of Sciences, Hungária Krt. 21, Budapest H-1 143, Hungary

## Abstract

*Picobirnavirus* (PBV) which has been included in the list of viruses causing enteric infection in animals is highly versatile because of its broad host range and genetic diversity. PBVs are among the most recent and emerging small, nonenveloped viruses with a bisegmented double-stranded RNA genome, classified under a new family “*Picobirnaviridae*.” PBVs have also been detected from respiratory tract of pigs, but needs further close investigation for their inhabitant behavior. Though, accretion of genomic data of PBVs from different mammalian species resolved some of the ambiguity, quite a few questions and hypotheses regarding pathogenesis, persistence location, and evolution of PBVs remain unreciprocated. Evolutionary analysis reveals association of PBVs with *partitiviruses* especially fungi *partitiviruses*. Although, PBVs may have an ambiguous clinical implication, they do pose a potential public health concern in humans and control of PBVs mainly relies on nonvaccinal approach. Based upon the published data, from 1988 to date, generated from animal PBVs across the globe, this review provides information and discussion with respect to genetic analysis as well as evolution of PBVs of animal origin in relation to human strains.

## 1. Introduction

Since the first detection of small sized bisegmented double-stranded RNA (ds-RNA) virus named* Picobirnavirus* (PBV) in humans and black-footed pigmy rice rats in 1988 [1, 2], it has been identified in various domestic and captive animals. The sequencing of partial segment 1 and full length segment 2 of this virus by Rosen et al. [3] unraveled some of the mysteries regarding its genome. Though accretion of genomic data of PBVs from different mammalian and reptile species across the world resolved some of the ambiguity, quite a few questions and hypotheses regarding pathogenesis, persistence location, and evolution of PBVs remain unreciprocated. The* Picobirnavirus* with main emphasis on its biology, epidemiology, viral persistence, and their zoonotic potential has been reviewed recently [4, 5]. Based upon the published data, from 1988 to date, generated from animal PBVs across the globe, this review provides information and discussion with respect to genetic analysis as well as evolution of PBVs of animal origin in relation to human strains.

## 2. Taxonomy, Classification, and Nomenclature

As the PBV has bisegmented genome revealed in polyacrylamide gel electrophoresis (PAGE), it was initially thought to belong to family* Birnaviridae*. Nevertheless, based upon differences from members of* Birnaviridae* with respect to host, virion size, capsid, RNA polymerase, genome size, and organization, the virus has been classified distinctly (Table 1). A new viral family named* Picobirnaviridae* under the proposed order “*Diplornavirales*” was created to accommodate this unique virus and a complete new taxonomic order was assigned (http://www.ictvonline.org/virusTaxonomy.asp). This new viral family is composed of only one viral genus,* Picobirnavirus*. The two species under the genus are Human* Picobirnavirus* and Rabbit* Picobirnavirus*, where the former one is nominated as a type species and the latter one as designated species by the International Committee on Taxonomy of Viruses in 2008 [6] (Taxonomy of* Picobirnavirus* list is as follows.) The nomenclature of the virus has been derived from its size and genome characteristics: the prefix “pico” signifies the small diameter of the viral particle (35 nm) and “birna” signposts a genome composed of two segments of dsRNA [2].

 Family:* Picobirnaviridae*
 Genus:* Picobirnavirus*
 Type species: Human* Picobirnavirus*
 Designated species: Rabbit* Picobirnavirus*
 Unassigned isolates:
   Bovine* Picobirnavirus*
   Equine* Picobirnavirus*
   Pig* Picobirnavirus*
   Dog* Picobirnavirus*
   Chicken* Picobirnavirus*
   Guinea pig* Picobirnavirus*
   Rat* Picobirnavirus*
   Giant anteater* Picobirnavirus*
   Hamster* Picobirnavirus*
   Snack* Picobirnavirus*.

Based on the RNA-dependent RNA polymerase (RdRp) gene (segment 2) of human PBV, the viruses are classified into two genogroups, that is, genogroup-I (G-I) [reference strain-1-CHN-97] and genogroup-II (G-II) [reference strain- 4-GA-91] [3, 7]. Remarkably, to date, out of 515 PBV sequences including both segments 1 and 2 available in the National Center for Biotechnology Information (NCBI), 83.11% are of genogroup I and only 2.52% are of genogroup II; however, the rest of them are undefined yet. In 2009, a uniform nomenclature for PBV was proposed which recommends the determination of genogroups (GI or GII), host, country of origin, strain, and year of isolation for a specific PBV identified [8]. For example, GI/PBV/human/China/1-CHN-97/1997 specifies a PBV with genogroup I specificity and strain name, 1-CHN-97, detected in human from China in the year 1997.

## 3. Virus Structure and Genome Properties

**Figure 1:**
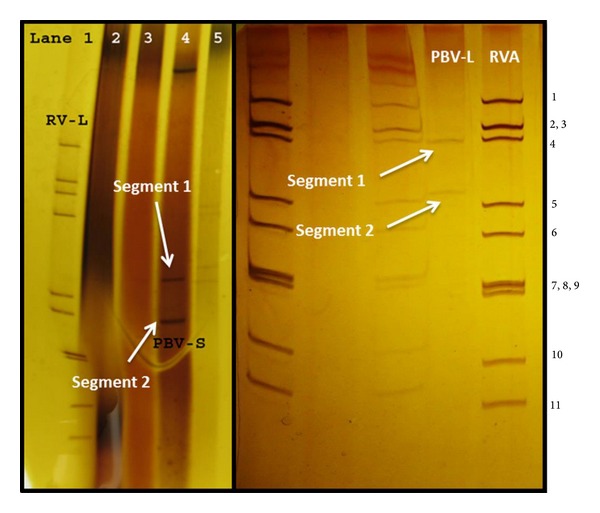
Silver stained polyacrylamide gel electrophoresis showing the bisegmented genome of* Picobirnavirus* (PBV-L; large genome profile of PBV, PBV-S; small genome profile) in comparison to group A rotavirus. RNA segments of group A rotavirus (RVA) are numbered according to the electrophoretic mobility in polyacrylamide gel.

PBVs are small (35–41 nm in diameter), non-enveloped, double-stranded, and bisegmented RNA viruses [2, 3]. Based on migration distance and size of segments 1 and 2, PAGE analysis with silver staining showed banding of genomic segments in two patterns, large and small genome profiles [5, 9–12]. In larger genome profile, the segments 1 and 2 correspond to 2.7 kb and 1.9 kb, respectively, while 2.2 kb and 1.2 kb, respectively, for short genome profile PBVs [5] (Figure 1).

The gene segment 1 (2.2–2.7 kb) encodes the capsid protein, while the gene segment 2 (1.2–1.9 kb) encodes the viral RNA-dependent RNA polymerase (RdRp) [13, 14]. The first 3.4 A° X-ray structure of a rabbit PBV in the form of virus like particles (VLPs) produced from open reading frame-2 (ORF-2) within segment 1 in* baculovirus* has been revealed recently [15]. The structure shows a simple core capsid with a distinctive icosahedral arrangement, displaying 60 two-fold symmetric dimers of a coat protein (CP) with a new 3D-fold. Like the most of the non-enveloped animal viruses, CP undergoes an autoproteolytic cleavage, releasing a posttranslationally modified peptide that remains associated with nucleic acid within the capsid. The capability of PBV particles to disrupt biological membranes* in vitro* has also been studied which indicates evolution of animal cell invasion properties of its simple 120-subunits capsid [15].

The analysis of three open reading frames-1 (ORF1) sequences (segment 1) available in databases representing three phylogenetically distant* Picobirnaviruses* (two from human: NC007026/human1 PBV [16] and GU968923/human2 PBV [17] and one from rabbit:* Picobirnavirus*, AJ244022/rabbit PBV [18]) were found to carry a particular sequence motif (ExxRxNxxxE) which is repeated four to ten times, depending on the virus strains and encoded proteins of various sizes (106–224 residues and without proline and cysteine) [19].

While conscripting this paper in 2013, only two full length PBV genome sequences were available in nucleotide sequences databases, that is, GI/PBV/human/THAI/Hy005102/2002 [16] and GI/PBV/California sea lion/Hong Kong/HKG-PF080915/2012 [20]. Complete nucleotide sequences of segment 1 of Lapine PBV [18] and segment 2 of bovine PBV [21] are also accessible.

### 3.1. Human* Picobirnaviruses*

**Figure 2:**
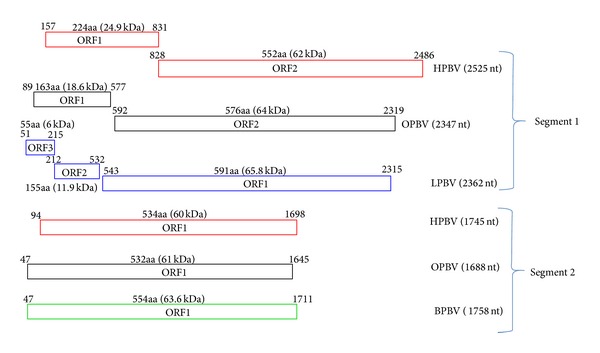
Comparison of open reading frames (ORFs) of* Picobirnaviruses* (segments 1 and 2) of different species. HPBV (Human* Picobirnavirus*), OPBV (Otarine* Picobirnavirus*), LPBV (Lapine* Picobirnavirus*), and BPBV (Bovine* Picobirnavirus*).

The segment 1 of Hy005102 strain is 2525 nt in length with GC contents of 45.8%. The 5′-non-coding region is AU rich (GC content: 36.5%) and a polyadenylation signal (AAUAAA) is absent. The segment 1 sequence has two long open reading frames (ORF1 and ORF2) (Figure 2). Two nucleotides, UG at positions 829 and 830, overlap as part of a termination codon for ORF1 and part of an initiation codon for ORF2, although the possibility of the occurrence of −1 frame shifting at this site cannot be excluded. ORF1 and ORF2 code for 224aa (24.9 kDa) and 552aa (62 kDa) proteins, respectively. The segment 2 of Hy005102 strain is 1745nt long with GC contents of 46.4%. The 5′-non-coding region is AU rich (GC content: 22.6%), as in segment 1, and five-nucleotide sequences, GUAAA at the 5′-end, are conserved in segments 1 and 2 [16].

The RdRp gene of prototype strains for genogrouping, that is, 4-GA-91 (genogroup II) and 1-CHN-97 (genogroup I), is 1674 nt and 1696 nt in length, respectively [3].

### 3.2. Otarine* Picobirnaviruses*

The segment 1 of PF080915 strain is 2347 nt long with GC contents of 42.8%. The 5′-non-coding region (88 bases) is AU rich (GC content of 40.9%), whereas the 3′-non-coding region (28 bases) has GC contents of 71.4%. It contains two open reading frames (ORFs), ORF1 and ORF2 (Figure 2). Segment 2 is 1688 nt long with GC contents of 47.45%. The 5′-non-coding region (46 bases) is also AU rich (GC content of 28.3%), whereas the 3′-non-coding region (43 bases) has GC contents of 46.5%.

### 3.3. Lapine* Picobirnaviruses*

The segment 1 of strain 35227/89 is 2362 nt in length [18]. The gene encodes three ORFs (Figure 2). The presence of stop codons at nucleotides 213–215 and 530–532 raises the possibility that two frame shifts may occur during translation to generate one long protein from nucleotides 51 to 2312.

### 3.4. Bovine* Picobirnaviruses*

The gene segment 2 of strain RUBV-P is 1758 nt long, with GC contents of 41.9% (Figure 2). The 5′-untranslated region is AU rich (78%) [21]. Interestingly, the 5′-(GUAAA) and 3′-(ACUGC) end sequences of gene segment 2 are conserved in the bovine strains and two human genogroup I PBV strains mentioned above.

## 4. Epidemiology and Impact on Health

**Figure 3:**
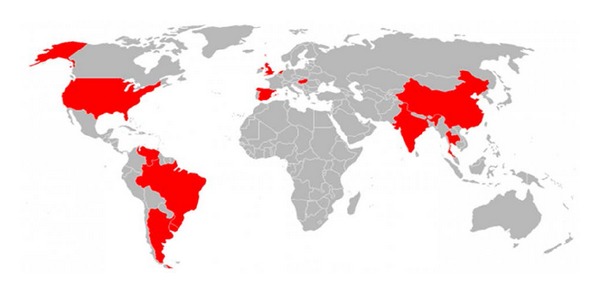
Global distribution of* Picobirnaviruses* (red colour shedding is done in those countries from where* Picobirnavirus* has been detected in any species including sewage).

**Figure 4:**
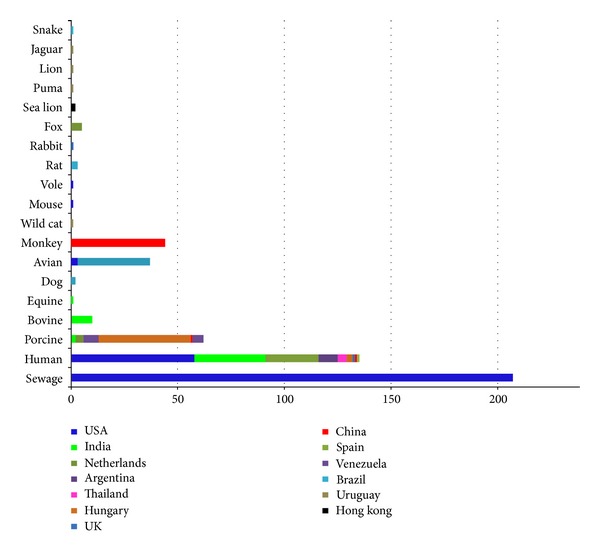
Species-wise distribution of* Picobirnaviruses* across the globe based on the nucleotide sequences of both segment 1 and segment 2 of PBV (either partial or full length) available in the NCBI database.

In efforts to detect causative agent from human suffering with gastroenteritis, Pereira et al. [1] for the first time detected PBV in the stool samples fortuitously. Thereafter, PBVs have been detected in the faecal samples of many animal species including rats [2, 22], chickens [23–27], hamsters [2], guinea pigs [28], pigs [29–37], bovine calves [10, 11, 21, 38, 39], water buffalo calf [12], foals [40, 41], snake [22], giant anteaters [42],* Panthera leo*,* Panthera onca*,* Puma concolor*, and* Oncifelis geoffroyi* [43]. Global and species-wise distribution of PBVs is presented in Figures 3 and 4, respectively. The PBV prevalence studies done so far in farm and captive animals across the world have been compiled and presented in Table 2.

The detection of PBV in various domestic and captive animals suggests that PBV has a wide host range. Initial studies carried out to develop an association of PBV with gastroenteritis yielded contradictory results. Gatti et al. [29] were of the first researchers to investigate the association of PBV with diarrhea in animals since Pereira et al. [1, 2] investigated this topic previously in humans and animals. Gatti and coworkers [29] screened 912 faecal samples of pigs in Brazil and detected PBV alone or as mixed infection with rotavirus in 15.3% diarrhoeic (rotavirus and PBV in 3.1%) and 9.6% in nondiarrhoeic pigs (rotavirus and PBV in 1.9%). Subsequent investigations by Ludert et al. [31] in Venezuela failed to show an association of* Picobirnavirus* infection with diarrhoea in contrast to Gatti et al. [29]. High incidence of PBV in pigs without diarrhea (12.3%) compared to pigs with clinical diarrhoea (10.0%) was reported with frequent detection (16.9%) in pigs aged 15 to 35 days. Similar type of studies in chickens revealed PBV incidence of 3.4% to 49.4% in the faecal samples or intestinal contents, more frequently in faeces with pasty consistency [23, 26, 27].

Notably, all the studies on etiology of PBV in captive animals presented lack of association of PBV with diarrhoea [2, 22, 42, 44–46]. The captive animals had no signs of diarrhoea or other evidence of enteric disease. During an extensive and systematic study carried out by Masachessi et al. [44] on 150 animals species in captivity at Córdoba city zoo of Argentina, PBVs were detected in different animals species like armadillo, donkey, orangutan, gloomy pheasant, pelican, and Chinese goose but none of them exhibited any signs of diarrhoea or enteric disease.

PBVs are most often isolated as coinfected agents with a number of diarrheal causes such as* Rotavirus* [47–50],* Astrovirus* [48, 49],* Caliciviruses* [7],* Escherichia coli* [51], and* Salmonella* [49]. These studies indicated that PBV might have played synergistic effect in association with the primary enteric cause. PBVs have also been identified in immunocompromised patients such as those infected with HIV [52–55]. Indication of concomitant infection having both the genogroups (GG-I and GG-II) of PBVs in one host has also been testified in humans [56], pigs [36], and more recently in bovines [11]. Unlike gastrointestinal tract, the normal or opportunistic inhabitant setting of PBVs, they were for the first time isolated from the respiratory tract of pigs with no evidence of overt respiratory or other diseases [36].

Atypical PBVs have also been detected in the oocysts of* Cryptosporidium parvum* from human stool samples [18, 57, 58] and in calves [38]. These viruses had smaller genome (two RNA segments are of 1786 bp and 1374 bp) and were highly consistent in their RNA electropherotypes [58, 59]. In contrast to those of typical PBVs, there is marked difference in coding specificity of these atypical PBVs in that segment 1 codes for viral RNA polymerase while segment 2 codes for a capsid protein.

The authors anticipated that captive animals might be acting either as the reservoir or persistent asymptomatic carriers, while in domestic animals PBV might be residing as opportunistic pathogen and different physiological conditions (age, lactation, pregnancy, and stress) assist in establishment of the infection [2, 22, 42, 44–46].

## 5. Laboratory Diagnosis

Peculiar bisegmented nature of PBV genome excluding* Birnaviruses* in animals had been exploited by many researchers for a long time for their diagnosis. Electron microscopy has been used for visualization of different animal PBVs [2, 23, 24, 30, 31, 39, 40, 42, 45, 47, 60, 61].

In the very first report of PBV dating back to 1988, it was detected in humans and black-footed pigmy rice rats [1, 2] accidently as the two migrated segments in PAGE. To date, direct visualization of PBV genome in PAGE after silver staining [62] has still been used in many parts of the world for reliable diagnosis. The PBV display at least two genomic profiles in PAGE, that is, large genome profile [segment 1: 2.3 to 2.6 kb and segment 2: 1.5 to 1.9 kb] and small genome profile [segment 1: 1.75 kb and segment 2: 1.55 kb]. In our studies, we came across the PBV of the larger genome profile in bovine specimen; on comparing the migration pattern with typical bovine rotavirus A, the larger band of PBV corresponded to segment 2 (2.6 kbp) while smaller band migrated up to the position between segments 4 (2.3 kbp) and 5 (1.6 kbp) of group A rotaviruses [12]. Notably, a third genome segment appeared in chicken [24] and dog [63]. These viruses with trisegmented dsRNA genome might be due to mixed infection of PBV strains or with other viruses; which, needs further investigation to confirm and/or ascertain the identification.

Keeping in account the poor sensitivity of PAGE, molecular based tests like reverse transcriptase-polymerase chain reaction (RT-PCR) was developed for the cloning and sequencing of the partial genome of two human PBV strains [3]. For genogrouping of PBVs, oligonucleotide primers targeting the RdRp gene are based on two prototype strains GI/PBV/human/China/1-CHN-97/1997 and GII/PBV/human/USA/4-GA-91/1991 (Table 3) and have been widely employed for genogrouping by RT-PCR [7, 9–12, 22, 34, 41, 49, 56, 64–66]. In our recent studies, we detected both genogroups in a bovine calf [11] and piglets (yet not published) testifying the utility of in-use genogrouping primers of PBVs. However, to further improve the diagnosis and identify the highly diverse porcine PBVs, diagnostic primers sets (PBV2-19 [+] 5′-CGACGAGGTTGATAAGCGGA-3′ and PBV2-281 [−] 5′-CACAGTTCGGG CCTCCTGA-3′) targeting conserved region of RdRp gene (824–1086 nt) allowed detection of porcine-like PBVs in humans [33]. Improved target set of oligonucleotide sequences for segment 2 based RT-PCR for bovine PBVs with high sensitivity and specificity has been developed (data not shown) and the same primer sets have also been found useful for detecting PBVs in pigs. However, for genogrouping of both bovine and porcine PBVs, published primers of Rosen [3] are quite satisfactory.

At present, animal model and permissive cell lines have not been recognized for PBVs which greatly hinders in their isolation and clinic-pathological studies.

## 6. Viral Persistence

So far, limited studies have been carried out to determine association of intermittent faecal shedding of PBV over a period of time by RNA-PAGE or RT-PCR with the persistence. Thus, the exact location, duration, and mechanism of persistence remain unsettled.

Oral infection of three newly weaned rabbits with purified PBVs led to excretion of maximum virus in faeces on day 13 [60]. Haga et al. [42] detected PBV weekly up to 4 months in three captive giant anteaters which did not show any signs of enteric disease during the study. They related their findings of prolonged shedding of PBV with the chronic infection which might be due to development of persistent infection. In another controlled experimental study conducted by Masachessi et al. [44], PBVs were detected by RNA-PAGE intermittently up to 6 and 7 months in captive armadillo and orangutan, respectively.

The use of RT-PCR combined with RNA-PAGE by Martínez et al. [35] provided better understanding about the ecological pattern of porcine PBV circulation in Argentina where follow-up studies were carried out from weaning (26 days after birth) to fourth reproductive cycle (898 days old) in female pigs. During the first week after weaning, PBV was detectable only by RT-PCR but, at 2 months, it could also be detectable by RNA-PAGE. Thereafter, intermittent episodes of PBV excretion were observed. Continuous PBV excretion pattern was identified in the first gestation and farrowing cycle and also during the third and fourth reproductive cycles; the rate of PBV detection was found maximum during the lactogenic period.

Recently, Masachessi et al. [67] provided the first evidence of persistent infection in birds (greater rheas) from Argentina. PBVs were excreted by these birds with nucleotide sequence identity between 90.5 and 100% in a longitudinal study with the possible involvement of single PBV strain with different electropherotypes profiles.

Together, these studies suggest the animals in their first week of life might acquire the PBV infection followed by establishment of persistent infection, with intermingled periods of high, low, and no virus detections depending on the age, season, and physiological status of the animals. The long term persistent within host could reasonably explain the higher genetic heterogeneity of PBV strains.

## 7. Phylogenetic Analysis and Evolution

**Figure 5:**
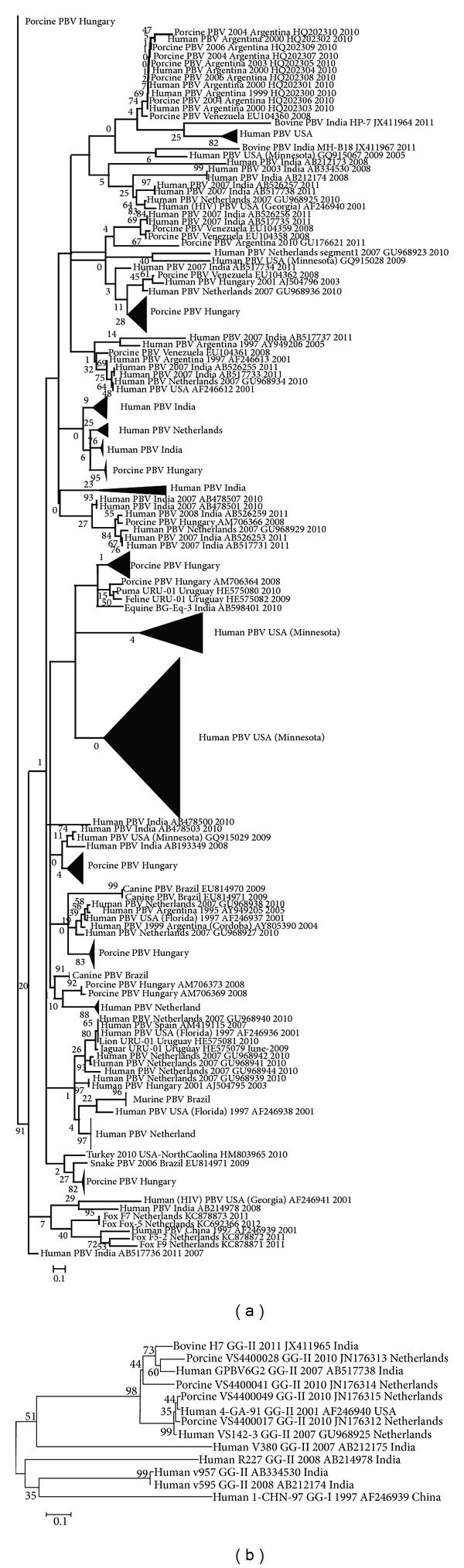
Phylogram showing genetic relatedness between animal and human* Picobirnaviruses* based on partial RdRp gene sequences retrieved from the GenBank database (http://www.ncbi.nlm.nih.gov/). (a) Genogroup I* Picobirnaviruses* of various species; (b) genogroup II* Picobirnaviruses* of human, porcine, and bovine origin. Phylogenetic tree was constructed by neighbor-joining (NJ) method implemented in MEGA5 (http://megasoftware.net/). Numbers on branches indicate percentages of bootstrap support from 2,000 replicates.

Sequence data retrieved from the GenBank database was phylogenetically analyzed by MEGA 5.05 software (http://megasoftware.net/). The PBV nucleotide sequences of different animal species were aligned using ClustalW with human PBVs along with GG-I and GG-II reference strains. The neighbor-joining statistical method using the maximum composite likelihood substitution model with 2000 bootstrap replicates was used for the construction of phylograms [68]. Close homology of animal PBVs with human PBVs is evident in the phylogram indicating the possible jumping across the species barrier (Figure 5(a)). The RdRp sequences comparison revealed sequence similarity >42% (at nucleotide level) and >40% (at amino acid level) for the different species of PBVs RdRp (GG-I) analyzed with human PBV GG-I reference strain (1-CHN-97) taking into account all the PBVs sequences accessible in NCBI database (Table 4). Notably, four human PBV strains (R227, V380, v595, and v957), though amplified by GG-II primers set (PicoB23 and PicoB24), displayed low sequence similarity with both human GG-I (1-CHN-97) [23.1–26.2% at nucleotide and 14.3–28.6% at amino acid levels] and GG-II (4-GA-91) reference strains [24.0–33.7% at nucleotide and 14.3–20.0% at amino acid levels] and were outgrouped away from the bovine, human, and porcine PBVs GG-II sequences (Figure 5(b)).

**Figure 6:**
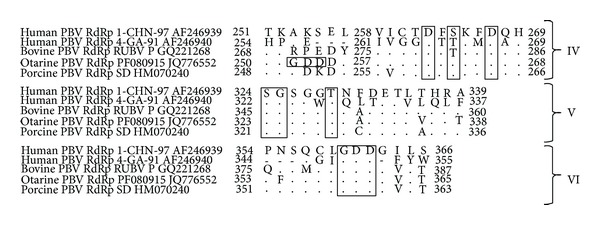
The aligned amino acid sequences of RdRp domains (IV–VI) of* Picobirnaviruses* with marked three conserved RdRp motifs (domain IV: D-S- -D, domain V: SGXXXT, and domain VI: GDD) representative of ds-RNA viruses in different animal species.

We also analyzed the RdRp gene of PBVs of different animal species and compared three conserved motifs residing in corresponding conserved domains of RdRp gene of ds-RNA viruses. The two motifs (SGXXXT and GDD) in domains V and VI, respectively, were found to be conserved in representative human, bovine, Otarine, and porcine PBVs (Figure 6). A single site difference was seen in third motif (D-S- -D) within domain IV in human GG-II and bovine PBVs where threonine replaced the existing serine in other PBVs. We found another GDD motif (252–254 aa) in otarine PBV upstream of domain IV (Figure 6).

**Figure 7:**
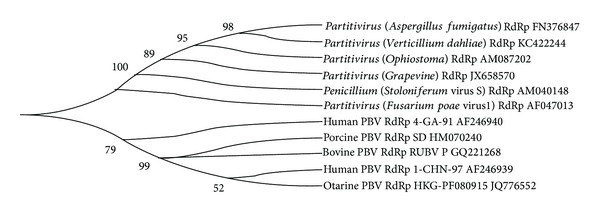
Genetic relatedness of different species* Picobirnaviruses* representatives with* Partitiviruses* of fungal origin based on full length RdRp gene (segment 2) sequences retrieved from the GenBank database (http://www.ncbi.nlm.nih.gov/). Phylogenetic tree was constructed by neighbor-joining (NJ) method implemented in MEGA5 (http://megasoftware.net/). Numbers on branches indicate percentages of bootstrap support from 2,000 replicates.

Since the appearance of PBV in 1988 in humans and black-footed pigmy rice rats and subsequently in various domestic and captive animals, evolution of these viruses is not well understood. One breakthrough in this respect came with the expression of capsid protein in the form of virus like particles (VLPs) in* baculovirus*. The analysis of PBV VLPs structure (made up of 60 symmetric dimers) showed that they are distinct from* Birnaviruses* and displayed a close relatedness with* Partitiviruses* (viruses infecting unicellular eukaryotes and plants) [69–71]. Since this close relatedness is due to capsid protein encoded by segment 1 of PBV, the authors analyzed the RdRp gene (segment 2) sequences of PBVs of different animal species with the* Partitiviruses* infecting fungi and plants. Comparative sequences analysis revealed the sequence similarity (18.6–22.0%) of different animals PBVs RdRp sequences with closely resembling fungal and plant* Partitiviruses* (Figure 7). It is interesting to note that the nucleotide similarity of PBV GG-I reference stain (1-CHN-97) with PBV GG-II reference stain (4-GA-91) is only 23.4%. The human PBV GG-I reference stain (1-CHN-97), human PBV GG-II reference stain (4-GA-91), bovine PBV (RUBV-P), otarine PBV (PF080915), and porcine PBV (SD) showed a maximum up to 20.6% nucleotide similarity with* Partitiviruses* of grapevine and* Aspergillus fumigatus*,* Aspergillus fumigatus* (22.0%), grapevine (bovine 21.1% and otarine 21.0%), and* Aspergillus fumigatus* (21.7%), respectively. The PBVs of animal origin made a separate cluster than* Partititviruses* (Figure 7).

These studies are suggestive of close relatedness of PBVs with* Partitiviruses* in respect to core protein and RdRp gene. It is hypothesized that during the course of evolution, it might be possible that these* Partitiviruses* had crossed the species barrier from fungi to vertebrates and got adapted or are adapting to the host they resided. Because of huge genetic diversity and outgrouping/separate clustering of some of the PBVs strains ascertain the needs to further extend the classification of PBVs into subgenogroups.

## 8. Interspecies Transmission

The crossing of the species barrier is defined in terms of genetic relatedness of one or more segments among the segmented genome viruses like* rotavirus* or PBVs infecting two different species from the same or different geographical areas. In case of PBV, a short fragment of RdRp gene was used most frequently in sequence comparison and phylogenetic analysis [9, 11, 12, 21, 33, 34, 41, 56]. Such studies on genetic relatedness were first carried out by Bányai et al. [34] between animal and human PBV strains from the same geographical area in Hungary where porcine PBV strain showed high sequence similarity (89.9% nt and 96.4% aa) with human PBV strain. Later, other studies described the genetic relatedness between human and porcine PBVs [9, 33, 37, 41], human and equine PBVs [56], and human and rodents PBVs [72]. In study of Ganesh et al. [9], four human PBVs (GPBV1-3 and 8) clustered with Hungary porcine PBVs (D4, D6 and C10). In another study by Ganesh [56], sequence comparison of a short stretch of the RdRp gene of equine PBV (BG-Eq-3) revealed close genetic relatedness (>98% nucleotide identity) to Indian human genogroup I PBV strain (Hu/GPBV1).

The detection of PBVs in sewage and surface waters [66, 73] at a relatively high frequency may further signpost the zoonotic potential of these viruses with emerging and/or re-emerging threat to a number of animals in different geographical locations (e.g., contamination of surface waters with runoff from animal feedlots). Extensive epidemiological studies are further needed to ascertain this observation. Extensive surveillance programs targeting this rapidly evolving and emerging virus in various species are indicated to understand its epidemiological pattern and zoonotic potential in different species in different geographical locations.

## 9. Conclusion and Future Perspectives

*Picobirnavirus* which has been detected in faeces of various animal species is highly versatile because of its broad host range and highly genetic diversity. RdRp gene based genogrouping (GG-I and GG-II) has helped in specifying viral genogroups circulating in animals across the different countries. The detection of PBVs from the respiratory tracts of pigs in addition to frequently gastrointestinal tract opportunistic inhabitant led to the expansion of knowledge on the tropism as well as host range. The studies revealed that PBVs are assumed to be acquired by the animals in their first week of life followed by establishment of persistent infection in undefined location and depending on the age, physiological conditions, and stress lead excretions/detection in the faeces. Probably PBVs might have evolved from the* Partitiviruses* especially fungi* Partitiviruses*. Replication strategies adopted by the virus and role of adaptive immunity has not been explicated so far. The close relatedness of animal PBVs with human along with detection of PBVs from the sewage designate the potential threat in terms of infection acquirement from the sewage and transmission of these viruses across the species. The outgrouping of some of the PBVs strains points to the need for further classification of PBVs into subgenogroups.

## Figures and Tables

**Table 1:** Key differences between *Birnaviruses* and *Picobirnaviruses*.

Properties	*Picobirnaviridae *	*Birnaviridae *
Hosts	Mammals	Fish, chicken, and turkey
Virion size (diameter)	35–40 nm	65–70 nm
Capsid structure	Triangulation of 1, 3 or 4	*T* = 13 laevo symmetry
RNA polymerase	A-B-C motifs	C-A-B motifs
Genome size	Smaller segment—1.7 kb and larger segment—2.5 kb	Smaller segment—2.8 kb and larger segment—3.3 kb
Genome organization (open reading frames)	Two or three overlapping ORFs (segment 1)	Single ORF

**Table 2:** *Picobirnaviruses* prevalence studies in domestic and captive animals (*PBVs isolated from respiratory tract).

Species	Total samples	RNA-PAGEpositive	RT-PCRpositive	Place of isolation	References
Porcine					
Porcine	912	11.6% (106/912)	—	Brazil	Gatti et al. (1989) [29]
Porcine	244	11.1% (27/244)	—	Venezuela	Ludert et al. (1991) [31]
Porcine	75	6.7% (5/75)	—	Canada	Alfieri et al. (1994) [47]
Porcine	557	0.4% (2/557)	—	Thailand	Pongsuwanna et al. (1996) [32]
Porcine	144	27.1% (39/144)	60.4% (87/144)	Venezuela and Argentina	Carruyo et al. (2008) [33]
Porcine	20	10% (2/20)	65% (13/20)	Hungary	Bányai et al. (2008) [34]
Porcine	265	21.1% (56/265)	—	Argentina	Martínez et al. (2010) [35]
Porcine∗	60	—	33.3% (20/60)	China and Sri Lanka	Smits et al. (2011) [36]
Porcine	11	18.2% (2/11)	18.2% (2/11)	India	Ganesh et al. (2012) [37]

Bovine					
Calf	576	0.7% (4/576)	—	Brazil	Buzinaro et al. (2003) [39]
Calf	136	3.7% (5/136)	—	India	Malik et al. (2011) [10]

Equine					
Horse	7	—	14.3% (1/7)	India	Ganesh et al. (2011) [41]

Canine					
Dog	163	1.8% (3/163)	—	Brazil	Costa et al. (2004) [74]
Dog	349	0.9% (3/349)	0.6% (2/349)	Brazil	Fregolente et al. (2009) [22]

Lapine					
Rabbit	211	10.9% (23/211)	—	Venezuela	Ludert et al. (1995) [60]

Simian					
Monkey	92	2.2% (2/92)	47.9% (44/92)	China and USA	Wang et al. (2007, 2012) [45, 46]

Avian					
Chicken	120	14.2% (17/120)	—	Brazil	Alfieri et al. (1989) [23]
Chicken	378	3.4% (13/378)	—	Brazil	Tamehiro et al. (2003) [26]
Chicken	85	15.3% (13/85)	49.4% (42/85)	Brazil	Ribeiro et al. (2014) [27]

Other species					
Mammals and birds	513	3.7% (19/513)	—	Argentina	Masachessi et al. (2007) [44]
Snake	82	8.5% (7/82)	2.4% (2/82)	Brazil	Fregolente et al. (2009) [22]
Rat	56	25% (14/56)	12.5% (7/56)	Brazil	Fregolente et al. (2009) [22]

**Table 3:** Oligonucleotide sequences for *Picobirnavirus* identification and genogrouping [3, 7].

Primers	Oligonucleotide sequences (5′-3′)	Genogroups	Amplicon size (bp)	Reference strains
PicoB25[+] (665–679)	TGG TGT GGA TGT TTC	Genogroup I	201	1-CHN-97
PicoB43[−] (850–865)	A(GA)T G(CT)T GGT CGA ACT T			

PicoB23[+] (685–699)	CGG TAT GGA TGT TTC	Genogroup II	369	4-GA-91
PicoB24[−] (1039–1053)	AAG CGA GCC CAT GTA			

**Table 4:** Percent identity of different animal species *Picobirnaviruses* with reference strains, GG-I (1-CHN-97) and GG-II (4-GA-91) at both nucleotide (NA) and amino acid (AA) levels.

Species	1-CHN-97 (GG-I reference strain)		4-GA-91 (GG-II reference stain)	
	NA (%)	AA (%)	NA (%)	AA (%)
Human (GG-I)	47.3–68.3	40.7–74.2	24.8–34.4	17.1–32.7
Human (GG-II)	21.1–31.2	15.3–27.8	47.6–68.0	55.3–74.9
Bovine (GG-I)	44.2–57.4	53.7–61.0	21.1–27.9	14.7–21.6
Bovine (GG-II)	23.1–24.2	16.2–19.0	48.8–67.0	55.5–66.7
Porcine (GG-I)	46.4–74.1	50.0–67.9	26.0–32.9	14.4–26.7
Porcine (GG-II)	26.0–27.4	14.4–16.1	62.2–97.9	72.1–99.1
Equine	62.4	66.1	28.8	19.6
Canine	53.0–64.3	57.6–72.3	30.6–31.5	24.2–24.6
Avian	55.7–65.7	59.1–72.7	27.4–33.2	21.2–33.1
Otarine	56.3	63.6	25.1	16.8
Mouse	53	60.6	22.6	16.6
Monkey	42.5–69.4	46.0–76.8	25.0–32.4	15.5–21.4
Fox	55.0–66.0	63.6–74.2	22.0–30.4	17.4–25.8
